# Detection of *Parvovirus* 4 in Iranian patients with HBV, HCV, HIV mono-infection, HIV and HCV co-infection

**Published:** 2018

**Authors:** Hosna Rastegarpouyani, Seyed Reza Mohebbi, Seyed Masoud Hosseini, Pedram Azimzadeh, Sedigheh beyraghie, Afsaneh Sharifian, Hamid Asadzadeh-Aghdaei, Shahnam Arshi, Mohammad Reza Zali

**Affiliations:** 1 *Basic and Molecular Epidemiology of Gastrointestinal Disorders Research Center, Research Institute for Gastroenterology and Liver Diseases Shahid Beheshti University of Medical Sciences, Tehran, Iran*; 2 *Gastroenterology and Liver Diseases Research Center, Research Institute for Gastroenterology and Liver Diseases, Shahid Beheshti University of Medical Sciences, Tehran, Iran*; 3 *Department of Microbiology and microbial biotechnology, Faculty of life Sciences and Biotechnology, Shahid Beheshti University, Tehran, Iran*; 4 *Foodborne and Waterborne Diseases Research Center, Research Institute for Gastroenterology and Liver Diseases, Shahid Beheshti University of Medical Sciences, Tehran, Iran*; 5 *Shahid Jafari HIV Reference Laboratory, Deputy of Health, Shahid Beheshti University of Medical Sciences, Tehran, Iran *

**Keywords:** Chronic infection, Hepatitis C virus, HBV, Parvoviridae, Parvovirus 4

## Abstract

**Aim:**

In this study, we investigated the prevalence of PARV4 virus among the healthy population and four other groups of HBV infected, HCV infected, HIV infected and HIV/HCV co-infected individuals in Iran.

**Background:**

*Parvovirus* 4 (PARV4) was first discovered in 2005, in a hepatitis B virus–infected injecting drug user (IDU). To date, the best evidence about PARV4 transmission is parenteral roots which comes from IDU individuals. It seems that the prevalence of the virus in the normal population is very low.

**Methods:**

A total of 613 patients, including chronic HCV (n=103), HBV (n=193), HIV (n=180) infected individuals, HIV/HCV (n=34) co-infected patients and 103 healthy controls, were studied by using nested-PCR and also real-time PCR techniques.

**Results:**

Of those 180 samples were positive for HIV RNA, co-infection of PARV4 was detected in 3 cases (1.66%). All these three patients were male with the age of 28, 32 and 36 years (mean: 32). No statistical differences were found between HIV positive group and the healthy individuals. (P>0.05) The result of PARV4 PCR was negative in all other samples and healthy controls as well.

**Conclusion:**

This study is the first to investigate the occurrence of PARV4 among these groups in Iran. The results show that the virus is not significant in Iranian population, even in patients with blood born infections such as HCV, HBV or even HIV patients. Further studies in other areas and various groups are required.

## Introduction

 Human *Parvovirus*-4 (‘PARV4’) is a member of *Parvoviridae *family which are known as single-stranded DNA viruses (1). Although the virus was discovered in 2005, there is not much knowledge about its prevalence, transmission, and clinical features (2).

There are currently three known genotypes of PARV4 (3) of which genotypes 1 and 2 are widespread in Northern Europe, the United States and Asia, and genotype 3 has been detected in sub-Saharan Africa (4-8). This virus seems to be transmitted parenterally since it was mostly detected in injection drug users (IDUs), hemophiliacs, and HIV- or HCV-infected patients (4, 5, 9). However, viral DNA detection in nasal and stool samples of children demonstrate that PARV4 may be transmitted among humans in respiratory or even foodborne by consuming contaminated food like foodborne viruses (10-12).

Despite the high prevalence in some populations, little is known about the routes or risk factors for transmission, host immune responses, the prevalence of viremia, or clinical impact of PARV4. (13) The virus has the potential to cause fever, fetal loss and chronic immunosuppression in pigs. In a recent study, it was suggested that like other viruses, PARV4 can cause severe human disease due to its interspecies jump, from animal to human (14, 15). On the other hand, the parenteral routes of transmission and the high prevalence in some population like injection drug users and hemophiliacs, indicate the importance of further studies on these viruses (14). 

There are many studies which are investigating the epidemiology of this virus. The outcome presents different distribution which demonstrates that PARV4 is endemic in some geographic areas. However, elsewhere, this distribution is restricted only to certain high-risk groups such as HIV positive individuals (2). 

In a recent study, PARV4 IgG was not detectable in 360 blood donors and control samples from the United Kingdom and France. In a Swedish study, there was no evidence of PARV4 in the severely immunocompromised HIV-negative children and adults. All patients had received frequent blood transfusions as well as other blood products (16). 

Another study in the UK demonstrated no PARV4 DNA in 624 respiratory samples from children <5 years of age (17). 

Different studies demonstrated 0–5% PARV4 DNA in healthy blood donors (5, 8, 12, 18, 19) and the frequency among IDU HIV positive individuals with AIDS have been reported up to 85% (9). Among HCV patients the frequency of co-infection varied from 8% to 30% (5, 9, 20, 21). 

The aim of this study was to investigate the prevalence of PARV4 among HIV, HBV and HCV mono-infected patients and HIV/HCV co-infected patients compared to healthy controls. Hypothesizing that viremia might be associated with age, sex or even viral co-infections. 

## Methods

**Samples**

In this study, we investigated 613 subjects of those who have referred to both Gastroenterology and Liver Diseases Research Center of Taleghani Hospital and Shahid Jafari HIV reference laboratory, Shahid Beheshti University of Medical Sciences, for the presence of the PARV4 virus. Our study subjects can be summarized in five different settings as follows:

I: HCV-positive patients (n=103): these samples were recruited from Gastroenterology and Liver Diseases Research Center, Taleghani Hospital (Tehran, Iran). We used Dia.Pro Diagnostic Bioprobes Kit (Italy, Dia.Pro) for HCV antibody detection.

II: HBV-positive patients who were HBsAg positive (n=193): these samples were divided into two groups: HBV DNA positive (n=103) and HBV DNA negative (n=90). All samples were recruited from Gastroenterology and Liver Diseases Research Center, Taleghani Hospital (Tehran, Iran). We determined HBsAg status using Dia.Pro Diagnostic Bioprobes Kit (Italy, Dia.Pro)

III: HIV-positive patients (n= 180): All samples were obtained from Shahid Jafari HIV reference laboratory, Shahid Beheshti University of Medical Sciences. 

IV: HIV/HCV co-infected patients (n=34) recruited from Gastroenterology and Liver Diseases Research Center, Taleghani hospital (Tehran, Iran). HIV 1,2 Antigen-Antibody Dia.Pro Kit (Italy, Dia.Pro) has been used for HIV antibody detection.

V: Healthy individuals (n=103) who were serologically negative for HBsAg, HCV antibody, and HIV antibody. The cohorts are available in Table 2. All subjects provided written informed consent for participation. 

Our focus was to detect the presence of PARV4 via molecular tests and we were unable to check the samples serologically due to the lack of commercial serology kits.

For each participant, 6 ml of blood was collected in an EDTA tube and the plasma was stored at −20 °C. The PARV4 status of these participants was unknown to us when they enrolled in the study. Samples were centrifuged at 3000rpm for 10 min, divided into cells and plasma and stored at −80 ◦C for further analysis. The details of the information related to sex and age of all individuals are presented in Table 2.

**Table 1:** Sequences of primers used in our experiment

No.	Primer Name		Primer Sequences	Amplicon size
1	PV4ORF1FPV4ORF1RPV4NS1Fn2PV4NS1Rn2	Round 1	5´- AAGACTACATACCTACCTGTG -3´5´- GTGCCTTTCATATTCAGTTCC -3´5´- GTTGATGGYCCTGTGGTTAG -3´5´- CCTTTCATATTCAGTTCCTGTTCAC -3´	220 bp
		Round 2		160 bp

DNA was initially extracted from pooled plasma samples using the QIAmp Viral DNA mini kit (Germany, QIAGEN), according to the manufacturer’ protocol. All samples were examined for PARV4 with polymerase chain reaction (PCR) and real-time-PCR. 

**Plasmids**

Synthesized plasmid containing PARV4 sequence was used for testing and optimization of the PARV4 real-time quantitative PCR. This sequence is conserved among different genotypes of virus including genotypes 1, 2 or 3. 

We chose a partial sequence driven by Gene Bank as a template and ordered to synthesize and clone that gene into vector pBSK-G driven of (+) pBLUSCRIP II (GeneRay Biotechnology, China). We prepared different concentrations of diluted plasmid and chose the lowest one as the positive control for screening tests. 

**Primers and hydrolysis probes**

For designing primers and probes for the two-step Nested PCR and qPCR, a sequence of ORF1 related to non-structural (NS1) protein of PARV4, combining both similarity and genotype-specific differences were selected based on a comprehensive PARV4 sequence alignment in GenBank. The primer sequences and locations in the PARV4 genome used in this study are presented in Table 1. The thermo-cycling conditions for nested PARV4 PCR in the first and second rounds were 95 °C for 9 min, followed by 40 cycles of 95 °C for 30 s, 55 °C for 30 s in the round 1 and 57 °C for 30 s in the round 2, and 72 °C for 1 min with a final extension of 7 min at 72 °C (22). PCR products were detected on 2% agarose gel (Agarose, Peq Gold universal, Belgium) and validated by sequencing. 

**Table 2:** The Demographic and Virological features of all samples (n= 613

	**HIV RNA** **positive %**	**HCV positive%**	**HBV DNANegative %**	**HBV DNA Positive %**	**HIV/HCV co-infected patients%**	**Healthy Controls %**
**Number**	180/613	103/613	90/613	103/613	34/613	103/613
**Sex (mean)MaleFemale**	68.3% 31.7%	60.2% 39.8%	54% 46%	52.5% 47.5%	90% 10%	41.7% 53.8%
**Age (mean)MaleFemale**	39.4±5.57 40.32±10.49	32.1±11.3 40.5±13.5	33.5±6.4 38.2±9.1	51.5±13.1 50.1±9.8	34.5±8.7 42.2±10.1	42/13±12.71 39.2±9.9

**Sequencing and phylogenetic analysis**

PCR product direct sequencing was performed bi-directionally on an ABI PRISM 3130xl DNA sequencer machine (Applied Biosystems, Foster City, California) following the manufacturer’s instructions. The result was edited by Bioedit software version 5.0.9. To further characterize this partial NS1 sequence, it was aligned with corresponding regions of 27 other known sequences retrieved from GenBank database using ClustalX software. The GenBank accession numbers for the PARV4 reference sequences used in the present study were as follows: HQ593530, HQ593532, EU175855, EU175872, EU546204, EU546205, EU546206, EU546207, EU200667, EU175856, EU546210, EU546211, EU874248, NJ798195, NJ798196, DQ873387, DQ873388, DQ873389, DQ873390, DQ873391, NC0070018, AY622943, KU871314, KJ541120, KJ541121 and GU120196. 

Chimpanzee parv4 isolate PT-P48 (GenBank accession number HQ113143) was utilized as an out-group. The result of multiple alignment were subject to phylogenetic analysis by MEGA 6 software (http:// www.megasoftware.net/), and the genotype was determined using the neighbor-joining method.

## Results

To investigate the frequency of PARV4 in high-risk groups and healthy controls, we analyzed previously collected serum samples from 510 patients and 103 healthy donors. The demographic data of all patients are shown on Table 2. 

Of those, 180 samples were positive for HIV RNA, co-infection of PARV4 was detected in 3 cases (1.66%). All these three patients were male with the age of 28, 32 and 36 years (mean: 32). No statistical differences were found between HIV positive group and the healthy individuals. (P>0.05)

**Figure 1:**
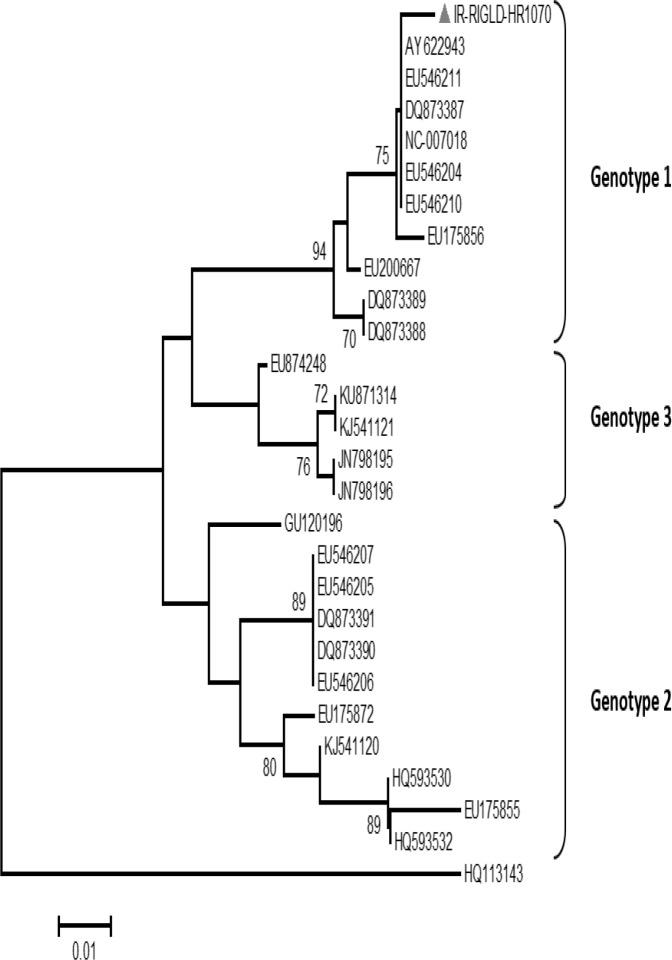
Phylogenetic tree of PARV4 sequences constructed based on 161 base pairs sequences ORF1 (NS1 protein region) using Neighbour joining method by Mega 6 software. Sequencing of a PARV4 positive sample from one Iranian patient (grey triangle) was performed and the result was compared to twenty seven reference sequences retrieved from GenBank which shown by their accession numbers

The result of PARV4 PCR was negative in all other samples and healthy controls as well. The PCR product of one PARV4 DNA was subjected to sequencing with forward and reverse primers. According to the result of phylogenetic analysis, the Iranian isolate was determined as a member of PARV4 genotype 1 and clustered with reference sequences of Genotype 1 accession numbers: AY622943, EU546211, DQ873387, NC0070018, EU546204, EU546210 and EU175856 (Figure 1).

## Discussion

This study is the first to investigate the occurrence of PARV4 among the healthy population and four other groups of HBV infected, HCV infected, HIV infected and HIV/HCV co-infected individuals in Iran. Many studies have been conducted on the epidemiology and various co-infections in the studied groups of Hepatitis B, Hepatitis C and also HIV infected patients, in Iran. (23-29). The frequency of PARV4 DNA in HIV infected individuals in our study was 1.66%. We found no evidence of PARV4 in the other groups. All three positive samples were determined as a member of PARV4 genotype 1 as it was reported in other studies on Iranian population (15, 30).

 Our result is in line with a study in Denmark on HIV-infected children, which 8.7 % of samples had detectable IgG and one had IgM. However, the result of polymerase chain reaction for PARV4 DNA detection was negative (31). In another reported study from the United Kingdom and France, PARVG IgG was not found in 360 blood donors and control samples (31). PARV4 DNA was detectable in 7 samples out of 113 (6.2%) of the HTLV-1-infected patients in Brazil.(32) 

Studies of children from Sweden, Finland, and the UK, Danish mother/child cohort also did not detect PARV4 DNA in the serum, tissue biopsies, or respiratory samples (16, 17, 33-35). However, PARV4 DNA has been found in blood samples of 2-9% of 15-months-old children in Ghana.(36, 37) In an African cohort, despite the high sero-prevalence of PARV4 (42.4%) in a total of 695 subjects included of HIV positive and HBV positive samples, PARV4 DNA has been identified in just 5 cases. (13) In a study from Taiwan, more than 50% of HIV-positive patients had IgG antibodies toward PARV4 (38). High frequency of PARV4 in HIV-infected individuals might be because of shared routes of transmission and parenteral exposure, as it was reported in some studies of injection-drug users (39). These reports support the hypothesis of the inconsistent epidemiology of PARV4 viruses among different populations. 

In blood donor subjects, the highest rate of positive serological results was observed in Burkina Faso and Democratic Republic of the Congo. These samples have been reported negative for HCV antibodies, and also HIV-1 antibodies, by third-generation screening. This report of high seropositivity rate among HCV screen-negative samples provides an evidence for an alternative route of PARV4 transmission which is largely absent in Western countries (40).

In Iran a few studies have been conducted on PARV4 viruses. Asyabi et al., reported PARV4 DNA in 35.3% of HIV infected individuals and 16.6% of healthy controls (15). However, in another study lower rate of infection has been reported by Amirahmadi et al.. 90 HBV patients and 90 healthy controls were tested to screen PARV4 DNA and the results showed the frequency of 4.4% among HBV samples and 1.1% of healthy controls (30). In 2017, Javanmard et al. reported a frequency of 9.3% of PARV4 DNA in patients with hemophilia (41).

As mentioned in a study conducted by Philippa C. Matthews and colleagues, differences in epidemiology of this virus among developed and developing countries is left unexplained and virus properties or even host characteristics like the host genetic differences, co-infection with other viruses, or other possible environmental factors might be the possible cause of such differences between countries (2). 

The clinical importance of PARV4 is less obvious and clinical symptoms that might be related to PARV4 pathogenicity have been found in a few reports. In a study, it was suggested that PARV4 may cause encephalitis in children because of the high viral load in cerebrospinal fluid and positive IgM and negative IgG titers in their serum samples. (42) PARV4 genotype 3 also detected in nasal and fecal samples of (0.83%) and (0.53%) of children <15 years in Ghana, respectively (43). In a study from Italy, PARV4 was suggested to have a role in liver and heart diseases. (39) In another study from Taiwan, Mao-Yuan Chen et al., reported PARV4 DNA in plasma from mothers and their newborns with hydrops to propose the possible transmission through the placenta (7). It was mentioned in a study that PARV4 may have an influence on the pathogenesis of HIV and HCV. (44) Overall, the clinical importance of PARV4 infection is still unclear. However, since these viruses are known as blood-borne viruses, they may cause some clinical symptoms, particularly in immunocompromised patients (2).

There would be many reasons to investigate on PARV4 Virus even if these viruses are not significant clinically. As an example, using these viruses as a vector to design new vaccines would be an acknowledgment of these reasons for the further investigation (2). It seems that the virus is not significant in Iranian population, even in patients with blood born infections such as HCV. However, further studies enrolling large number of different study subjects and collecting detailed diagnostic data, would be required to expand on this investigation.
